# The Rules of Aggression: How Genetic, Chemical and Spatial Factors Affect Intercolony Fights in a Dominant Species, the Mediterranean Acrobat Ant *Crematogaster scutellaris*

**DOI:** 10.1371/journal.pone.0137919

**Published:** 2015-10-07

**Authors:** Filippo Frizzi, Claudio Ciofi, Leonardo Dapporto, Chiara Natali, Guido Chelazzi, Stefano Turillazzi, Giacomo Santini

**Affiliations:** Department of Biology, University of Florence, Via Madonna del Piano 6, 50019, Sesto Fiorentino (FI), Italy; Salford University, UNITED KINGDOM

## Abstract

Nest-mate recognition plays a key role in the biology of ants. Although individuals coming from a foreign nest are, in most cases, promptly rejected, the degree of aggressiveness towards non nest-mates may be highly variable among species and relies on genetic, chemical and environmental factors. We analyzed intraspecific relationships among neighboring colonies of the dominant Mediterranean acrobat ant *Crematogaster scutellaris* integrating genetic, chemical and behavioral analyses. Colony structure, parental relationships between nests, cuticular hydrocarbons profiles (CHCs) and aggressive behavior against non nest-mates were studied in 34 nests located in olive tree trunks. Bayesian clustering analysis of allelic variation at nine species-specific microsatellite DNA markers pooled nests into 14 distinct clusters, each representing a single colony, confirming a polydomous arrangement of nests in this species. A marked genetic separation among colonies was also detected, probably due to long distance dispersion of queens and males during nuptial flights. CHCs profiles varied significantly among colonies and between nests of the same colony. No relationship between CHCs profiles and genetic distances was detected. The level of aggressiveness between colonies was inversely related to chemical and spatial distance, suggesting a ‘nasty neighbor’ effect. Our findings also suggest that CHCs profiles in *C*. *scutellaris* may be linked to external environmental factors rather than genetic relationships.

## Introduction

Recognition of nest-mates is essential in the biology of social organisms to preserve group cohesion and stability, which depends on privileged relationships among individuals of a social group [1–4]. In ants, the capacity of discriminating nest-mates is well developed and individuals coming from a foreign nest are, in most cases, promptly rejected [4–7]. The degree of aggressiveness towards non-nest mates may, however, be highly variable among species. There are examples, as in unicolonial ants, where intra-specific aggression is reduced or nonexistent and members of different nests can merge to form ecologically dominant “supercolonies” [8, 9].

Nest-mate recognition is based on a number of mechanisms, from visual identification to chemical communication, depending on the species involved [10–13]. Ants usually rely on chemical cues [14–19]. The chemical compounds produced by individuals are transferred among nest mates by trophallaxis, allogrooming or simple contact, and the resulting chemical mix contributes to the creation of a colony-specific odor template [20–23]. Cuticular hydrocarbons (CHCs) are particularly important compounds among the substances used by ants in nest-mate recognition [19, 24–28]. Genetics and environmental factors linked or somehow responsible for changes in CHCs profiles can also play a role in interindividual recognition [29–33]. In *Temnothorax longispinosus*, for example, genetic relatedness between colonies may be one of the main factors determining aggressive behavior [34]. On the other hand, nest-mate recognition in *Linepithema humile* appears to be highly dependent on environmental cues including diet [35, 36]. Workers can also learn the chemical profile of neighbor colonies [37, 38], and this may influence aggressive responses during subsequent encounters [39–41]. The relative importance of chemical, genetic and environmental parameters in conspecific recognition is nevertheless still a matter of debate [42].

In this study, we analyzed colony structure and assessed the potential role of genetic, chemical and spatial variables in nest-mate recognition in the acrobat ant *Crematogaster scutellaris* (Olivier 1792). Colonies of *C*. *scutellaris* are commonly found in both natural and human-managed ecosystems across the Mediterranean Basin [43, 44]. Nests are excavated in tree trunks or logs and can host up to several thousand workers [43, 45]. Previous work showed that *C*. *scutellaris* is one of the most highly ranked competitors in Mediterranean ant assemblages and has a pivotal role in community dynamics [46–49]. Preliminary evidence based on behavioral observations suggests monogynous reproduction, a polydomous nest arrangement and an inter-nest aggressiveness which varies with distance [50–53]. We investigated genetic structure of colonies in a human-managed agroecosystem using species-specific microsatellite loci. We then assessed occurrence of polydomy and parental relationships among different colonies and evaluated whether a correlation exists among genetic relationships, CHCs profiles and spatial distances among colonies. Finally, we conducted aggression tests between ants from different nests and compared aggression probability to chemical, genetic and spatial distance values. The study was intentionally conducted at a small spatial scale (34 nests over an area of approximately 1 hectare) in order to observe colonies that had a good probability to interact. The ant *C*. *scutellaris* plays a pivotal role in the ecology of Mediterranean arthropods. The study of behavioral mechanisms regulating the spatial arrangement of *C*. *scutellaris* colonies is therefore of significant importance for a more comprehensive understanding of the dynamics of ecological communities in the Mediterranean basin.

## Methods

### Study area and nest sampling

We conducted the study in an olive orchard near Florence, Italy, from March 2009 to September 2010. ‘Fattoria di Travalle’ owners gave us permission to work on their property. Climate of the study area is typically Mediterranean with hot summers and mild, wet winters. The orchard extended for approximately 40 ha on a hill side. Trees were distributed following a regular spacing planting design at intervals of about 6 meters. In total, 638 trees were surveyed over an area of approximately 3.5 hectares. Presence of *C*. *scutellaris* nests was assessed by repeated hammering on the trunk. A trunk was scored as hosting a nest when a defensive swarming of ants was elicited from nest holes. Ants were sampled within 50 cm from the nearest nest and single ants scouting on trees were not considered. Olive trees were the only nesting sites of *Crematogaster* ants in that area. Tree location was recorded on a map and we defined as “tree spatial clusters” groups of ant-hosting trees with no trees in between that did not host an ant nest [52]. We identified a total of 37 spatial clusters and we defined the size of each cluster as the number of nests found in the olive trees included in the cluster. Only one nest was found in each olive tree.

**Fig 1:**
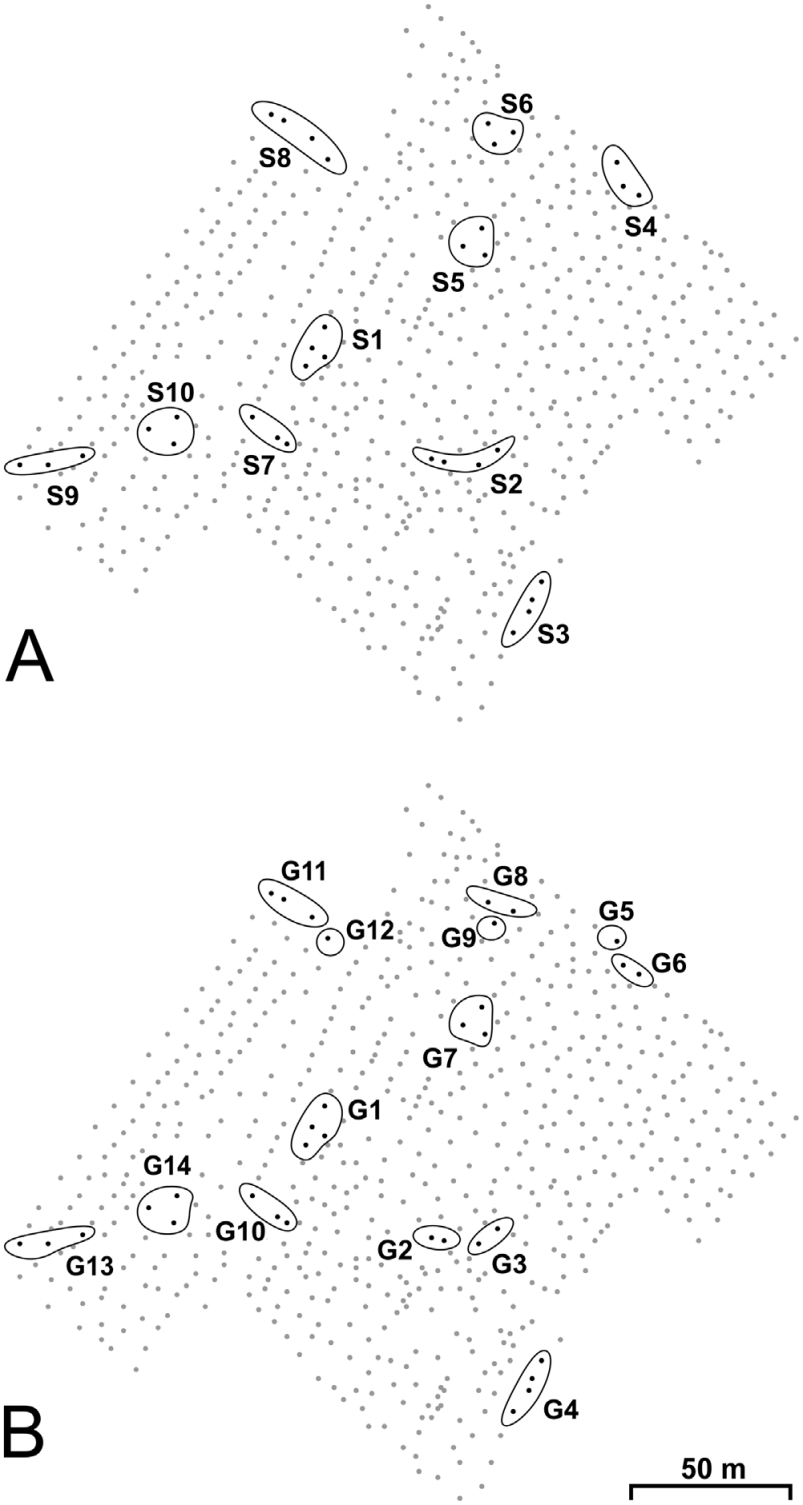
Spatial distribution of nest clusters in the study area. Dots are olive trees. Ovals represent nest clusters. A) spatial clusters (S1–S10); B) clusters resolved by genetic analysis (G1–G14).

A subsample of 10 spatial clusters, included within an area of approximately 1 hectare, was randomly chosen for genetic analysis, for a total of 34 nests. We selected also 29 of these nests for chemical analysis (five nests were apparently abandoned by ants between the first and second sampling, three weeks later). Six clusters had three nests each, while four nests were present in each of the remaining four clusters (Fig 1A). Distances between nests ranged from 4.8 m to 175.5 m, and there was no contact among branches of different trees. That precluded direct dispersal of ants between olive trees. We randomly collected six ant workers from each nest for a total of 204 and 174 ants screened for genetic and chemical variation, respectively.

### Genetic analysis

Ants were placed in 90% ethanol and subsequently stored at -80°C. Individual ants were ground in a microcentrifuge tube containing 300 μl 10% CHELEX 100 resin (BIO-RAD) and incubated at 95°C for 20 min. Samples were vortexed thoroughly, centrifuged for 15 s at 13,000 rpm, and 1 μl of supernatant was used for subsequent polymerase chain reaction (PCR) amplification. Allelic variation was assessed at nine microsatellite loci following multiplex PCRs as described in Ref [54]. PCR products were resolved on an ABI PRISM 3130xl genetic analyzer and allele sizes scored against a GeneScan 500 ROX size standard using GENEMAPPER 5.0.

Genetic diversity was quantified by measuring the average number of alleles per locus (allelic diversity), observed heterozygosity (*H*_O_) and gene diversity (*H*_E_) using FSTAT 2.9.3.2 [55]. Nest clusters were defined using a Bayesian clustering method implemented in BAPS 6 [56, 57] Clustering of molecular data and estimation of admixture proportions were performed at both individual and group level, where a group was defined as individual ants from the same nest. Posterior probabilities were assessed for a maximum of 34 clusters (the total number of sampled nests). Both types of analysis were repeated 10 times to ensure consistency of results between different runs. Changes in values of the logarithm of the Bayes factor obtained by moving individuals among all *k* clusters were computed to infer optimal clustering. The value is expected to be zero for the cluster where an individual is in the best solution. Small absolute values of the change (<2.3) indicate that an individual ant could be also allocated to a different cluster [58, 59] Genetic differentiation between genetic clusters and between tree nests within clusters was assessed by the *F*_ST_ estimator *θ* [60] using FSTAT 2.9.3.2. The effect of group type (clusters and nests within clusters) on genetic differentiation was assessed using a hierarchical likelihood-ratio *G*-test [61] implemented in the HIERFSTAT R-package [62] with the whole surveyed population as reference. We run 10,000 permutations of individuals both between nests within clusters and between clusters within the whole population. Statistical significance was assessed by 9999 random permutations. Finally, we inferred parentage among workers using a maximum likelihood method implemented in COLONY 2.0.5.0 [63].

### Chemical analysis

Workers from the same nest were kept alive in collection tubes in small groups of 20 or less individuals. Each ant was then separately frozen and stored at -80°C. CHCs were eluted from the exoskeleton of each ant by rinsing with 20μl heptane in a sample tube. A 2 μl solution was injected into a Hewlett Packard (HP) 5890A gas chromatograph coupled with a HP 5971A mass selective detector and a Zebron ZB-5 column (Phenomenex, CA) coated with 5% diphenyl–95% dimethyl polysiloxane. We used helium (12p.s.i., 82.7kPa) as carrier gas and set the injector port and transfer line at 300°C. The thermal protocol included a temperature ramp from 70°C to 150°C at a rate of 12°C min^-1^ with 2 min holding, and from 150°C to 320°C at 8°C min^-1^ with for 5 min holding. Analyses were performed in splitless mode. Cuticular compounds were identified on the basis of their mass spectra produced by electron impact ionization (70eV), and areas of the chromatogram peaks were then transformed into percentage values, which were subsequently used for statistical analysis. Chemical segregation between nests and nest clusters was evaluated by a non-parametric multivariate analysis of variance (npManova) performed using 999 permutations on the resulting pairwise distance matrix based on Bray-Curtis distances [64]. The possibility to attribute individuals to their group based in chemical components was assessed by Partial least squares Discriminant Analysis (PLS-DA, see Ref [65]) using mixOmics 5.0–3 R package. The analysis was performed considering both genetic clusters and nests as groups, with 13 and 28 components (number of groups minus one) returned by the model, respectively. A Mantel test based on the non-parametric Spearman correlation [66] was performed to estimate relationships between genetic, geographic and chemical distance values among nests from different clusters considered in the aggression tests (see below).

### Aggression tests

Aggression tests were performed using 5-to-5 battles between all possible pairwise combinations of nest clusters defined by genetic analyses [67, 68]. In each test, a cluster was represented by a single, randomly chosen nest. A total of 91 tests were conducted in the field from July to September 2010 at a distance of at least 30 m from the nearest nest. In order to control for inter-nest aggression due to manipulation stress, we performed 10 additional tests confronting ants collected from different nests of the same cluster. Ant groups of five individuals each were randomly collected from test nests and left undisturbed for 30 minutes in collection vials to avoid possible aggressive behavior induced by manipulative stress. Group pairs were then confronted in 5-cm diameter Petri dishes coated with Fluon® to prevent workers from escaping the arena. In polydomous species, aggressiveness towards non nest-mates can be affected by the presence or absence of the queen [69, 70]. However, nests of *C*. *scutellaris* are deeply excavated in the trunk, so that it was not possible to assess whether a nest hosted a queen. We observed ants for 5 consecutive minutes and recorded aggressive behaviors of single individuals during the entire test period. An individual ant was considered aggressive when showing clear signs of belligerent behavior, such as open mandibles, repeated and prolonged antennation or single/multiple bites. We then recorded the total number of aggressive ants. Behavioral tests were not completely blind for we knew which ants were obtained from different colonies. That might have resulted in the introduction of bias [71], however, the investigator had no knowledge of the degree of genetic or chemical relatedness between ants.

Results of aggression tests were modelled by Generalized Linear Models (GLM) with Poisson error term, using the R 3.0.2 statistical packages. We assessed the relationship between the number of aggressive individuals and genetic, chemical and spatial divergence for each pair of nests from different clusters. Models of different complexity were fitted to the data. The full model included chemical, genetic and spatial distances between each pair of nests involved in behavioral tests as explanatory variables. Models were compared using their AICc values (AIC values corrected for small sample size) and Akaike weights [72]. Several models showed similar AIC values. Coefficient values were therefore estimated from model-averaging. The importance of each predictor variable was then computed as the sum of the Akaike weights over models that included a particular variable [72]. Model-averaging computations were performed using the MuMIn R library.

## Results

### Genetic analysis

Allelic diversity ranged from 1.9± SE 0.10 to 2.8± SE 0.17 over 34 nests. Observed heterozygosity and gene diversity varied from 0.444± SE 0.059 to 0.911± SE 0.095 and from 0.176± SE 0.013 to 0.534± SE 0.035, respectively. Cluster analysis on individual and groups of individual ants defined a total of 14 clusters (G1 ÷ G14; Fig 1B). The majority of clusters included either two (G2, G3, G6 and G8) or three (G7, G10, G11, G13 and G14) nests. Single-nest clusters (G5, G9 and G12) and clusters made of four nests (G1 and G4) were also observed. Each worker was assigned to a single cluster with posterior probability *P* = 1 and in no case workers sampled on a given nest were assigned to a different one. Full correspondence between spatial and genetic clusters was observed in all but four of the original spatial clusters (S2, S4, S6 and S8; Fig 1A), each made of two genetically different clusters. Absolute values of changes of the log-marginal likelihood following reallocation of each individual ant from its cluster to other clusters ranged from 16.7 to 94.7. This value was equal to 0 for each individual belonging to its genetic cluster, indicating optimization in clustering accuracy.

Overall divergence among genetically defined clusters was high (mean *θ* = 0.400± SE 0.005). Pairwise *θ* values ranged from 0.251 (G6 vs. G9) to 0.524 (G11 vs. G14). Mean *θ* values between nests within clusters ranged from -0.026 (G1) to 0.050 (G2). The G-test showed no differences between nests within clusters (*P* = 0.244), while a statistically significant difference was recorded among clusters (*P* < 0.001). No correlation was recorded by Mantel test between genetic differentiation values and spatial distance (*r* = -0.149, *P* = 0.271).

Parentage analysis resulted in a single mother (queen) and a single father (male) for each genetically defined cluster, except for cluster G8 where a single queen and two different males were estimated with six workers fathered per male.

### Chemical analysis

**Fig 2:**
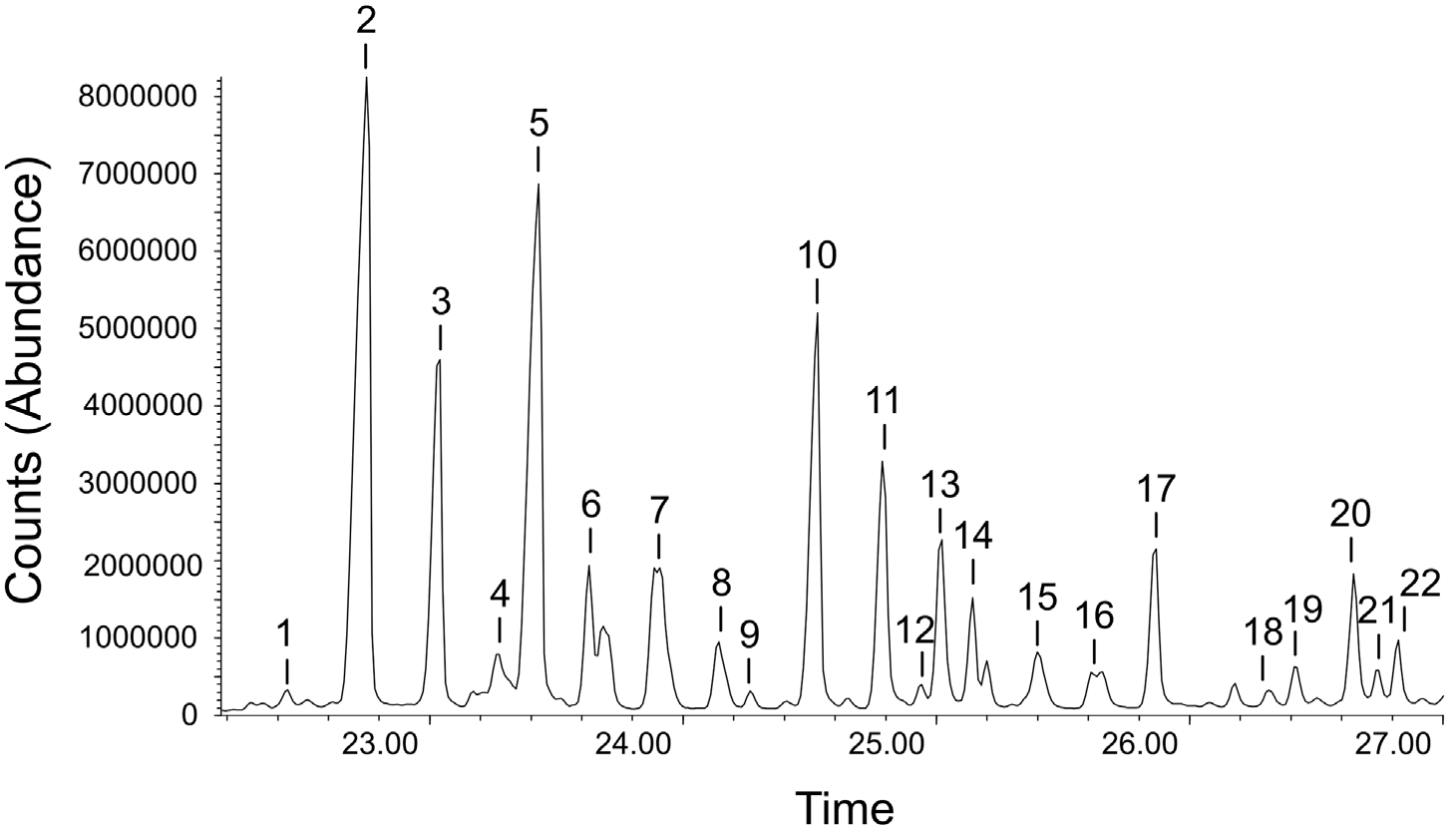
Example of a Gas chromatography-Mass spectrography (GS-MS) chromatogram of hydrocarbon molecules in *C*. *scutellaris*. The *x*-axis is the time (in minutes) elapsed from injection of the compound mixture to the elution of chemicals (retention time). The *y*-axis represents absolute abundance, which is related to the number of times each ion reported in the chromatogram struck the GS-MS detector. Chemical compounds are 1) c27_1, 2) c27, 3) M11c27, 4) DM11yc27, 5) M3c27, 6) c28, 7) CeMc28, 8) M2c28, 9) c29_1, 10) c29, 11) CeMc29, 12) M5c29, 13) DM11yc29, 14) M3c29, 15) c30, 16) CeMc30, 17) M4c30, 18) c31, 19) CeMc31, 20) DM11yc31, 21) DM7yc31, 22) DM5yc31. Compounds abbreviations: M: methyl; CeM: central-methyl; DM: dimethyl.

**Fig 3:**
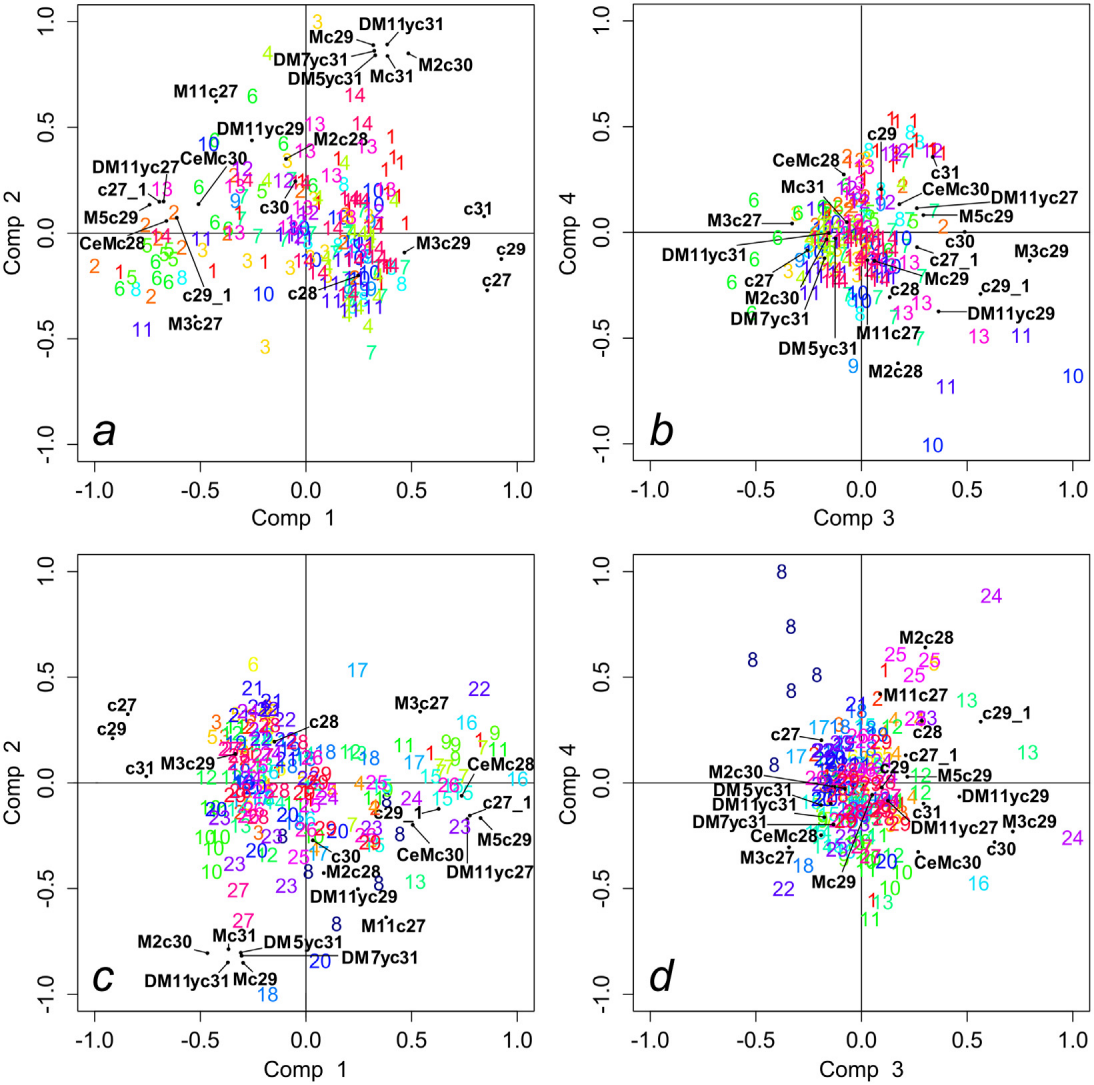
PLS-DA plots on chemical distances. Plots for genetic clusters (a, b) and nests (c,d) are described by the first two pairs of components (Comp). Scores were normalized between -1 and 1 and plotted in the same graph with the loadings of variables. Scores of individual ants of the same group are represented by the same number (14 genetic clusters and 29 nests). Loadings of variables are indicated by compound names (see Fig 2). According to biplot theory, proximity among cases (individuals) and variables (compounds) represents a tendency of individual ants to have a higher abundance of the studied compounds.

A total of 22 CHCs were recorded by GC-MS analysis in the chemical profile of the ants. An example of a GC-MS chromatogram is reported in Fig 2 (see Ref [73] for a description of cuticular signatures in *C*. *scutellaris*). The most abundant compounds were linear alkanes, monomethylated alkanes, dimetyhylated alkanes and alkenes. According to non-parametric Manova, chemical signatures of ants differed significantly among clusters and among trees within clusters (Table 1). A large overlap of data points was instead evident in all PLS-DA component scores plots (Fig 3). However, a number of groups could be differentiated depending on the components considered. An example is provided by group 8, which is clearly separated from other groups by PLS components 3 and 4 only (Fig 3d). No correlation was revealed by Mantel tests between either chemical and spatial distances (*r* = -0.0494, *P* = 0.728), or chemical distance and genetic differentiation values (*r* = 0.0525, *P* = 0.788).

**Table 1:** Results of non-parametric Manova on chemical distances among nests of different clusters and among nests within clusters.

Source of variation	d.f.	SS	MS	Pseudo-F	*P*
Among clusters	13	0.998	7.68E-02	6.386	**0.001**
Among nests within clusters	15	0.523	3.49E-02	3.026	**0.001**
Residuals	143	1.648	1.15E-02		

### Aggression tests

**Fig 4:**
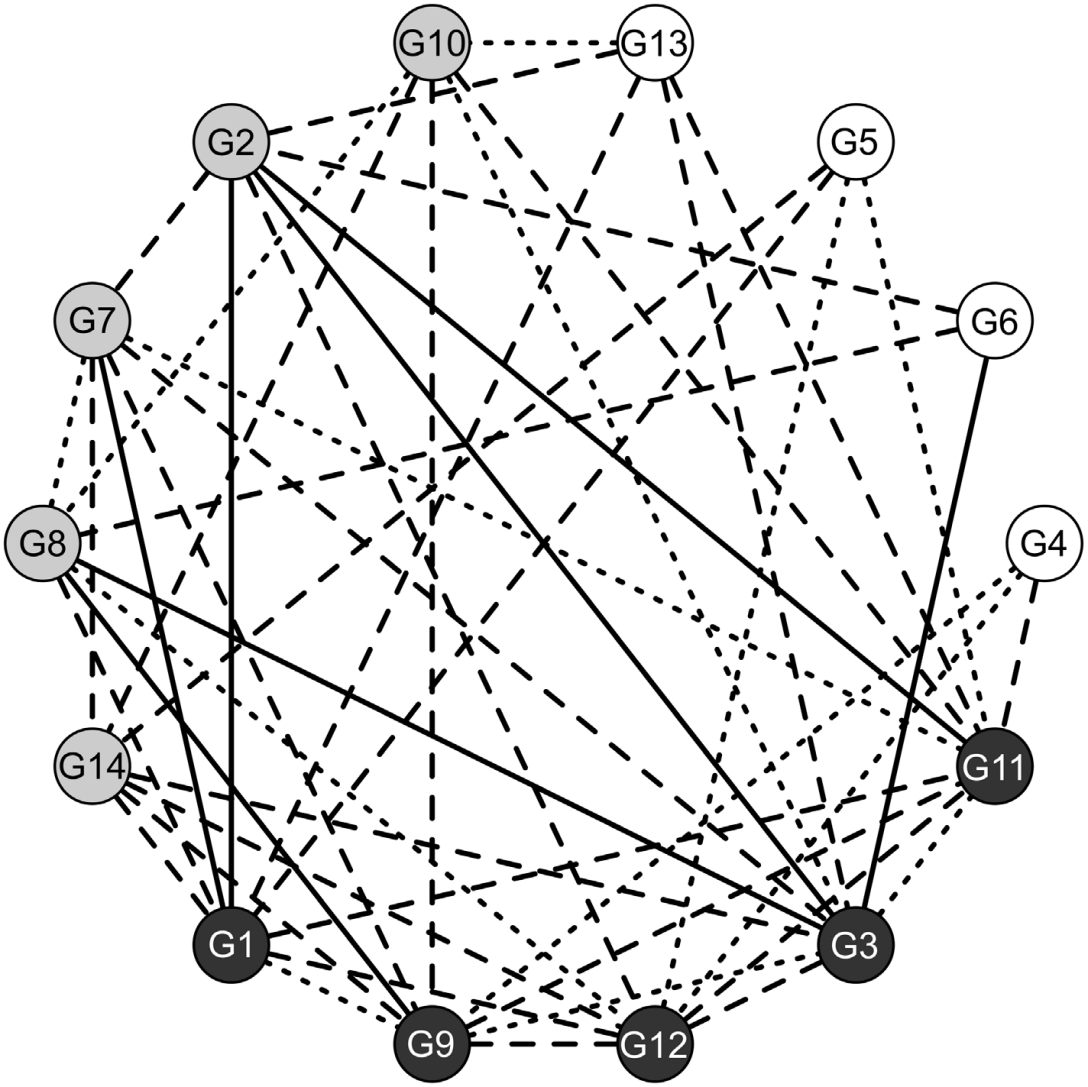
Schematic map of aggressive interactions between all possible pairs of nest clusters. Clusters are sorted according to the number of aggressive interactions they were involved in, clockwise from the most (G11) to the least aggressive (G4) one. Dark grey clusters showed aggressive behavior in more than half of staged tests. Light grey and white clusters showed signs of aggressions in half of the encounters and less than half of staged tests, respectively. Lines connecting nest clusters show levels of aggressions between each pair of clusters: no aggression (score 0, no line), aggressive display (score 1, dotted line), direct aggression (score 2, dashed line) and strong aggression (score 3, solid line).

No aggressive behavior (score 0) was observed during control tests. On the other hand, aggressive behavior occurred in 52.7% of staged tests. Aggressive displays (score 1) were observed in 13 encounters, while direct aggression (score 2) and strong aggression (score 3) occurred in 28 and 7 tests, respectively. No aggression or display behaviors were recorded in the remaining 43 tests. Ants from 5 clusters (G1, G3, G9, G11 and G12) showed aggressive behavior in more than half of the staged tests. In other 5 clusters (G2, G7, G8, G10 and G14) ants showed signs of aggressions in half of the encounters. In the last 4 clusters (G4, G5, G6 and G13) ants were aggressive in less than half of the tests (Fig 4). The most aggressive nests showed the largest proportion of strong aggression events. The number of aggressive events did not correlate with the number of nests forming a colony (Poisson GLM z value = -0.432, P>0.66). The models that best explained which factors may have influenced aggression probability included a) chemical, spatial and genetic distances and b) chemical and spatial distances (Table 2). However, there was a model selection uncertainty, given the small difference in AICc (ΔAICc<2) and similar Akaike weights among the first two models. Results of model averaging (Table 3) showed that aggression probability was negatively related to both chemical and spatial distances, but increased with genetic distance. According to the sum of Akaike weights, the most important variables were chemical and spatial distances (importance = 1.00 each) followed by genetic divergence (importance = 0.72).

**Table 2:** Results of GLM models assessing the relationship between the number of aggressive individuals and genetic, chemical and spatial divergences. Models are listed in order of increasing AICc value. Explanatory variables are chemical distance (chem), genetic *θ* values and spatial distance (spat) between each pair of nests. The difference between the AICc value of a specific model and the AICc of the best model (ΔAICc) is reported for each model considered in the regression analysis. W_i_ is the Akaike weight.

Model	AICc	ΔAICc	W_i_
chem + *θ* + spat	530.411	0.000	0.69
chem + spat	532.252	1.841	0.27
*θ* + chem	536.581	6.170	0.03
chem	539.725	9.314	0.01
*θ* + spat	550.112	19.701	0.00
spat	552.802	22.391	0.00
θ	556.545	26.134	0.00
null model	560.489	30.078	0.00

**Table 3:** Estimated coefficient values of model-averaging for the first two models listed in Table 2.

Coefficient	Estimate	SE	z	*P*
Intercept	2.117	0.658	3.192	**0.001**
chem	-5.613	1.259	4.397	**< 0.001**
spat	-0.005	0.002	2.897	**0.004**
*θ*	1.610	1.392	1.149	0.250

## Discussion

The distribution of *C*. *scutellaris* nests observed in this study was the result of a clear polydomous arrangement of colonies. Nearby nests mostly hosted genetically similar ants and were grouped together by genetic clustering analysis. The spatial arrangement of a colony over different nests may allow reduction of energetic costs involved in resource exploitation, whereby satellite nests are established in close proximity to essential resources [74, 75]. In the agroecosystem considered in our study, management practices such as mowing during summer time strongly reduce resources availability on the ground so that the majority of food items (i.e. prey insects and honeydew producing homopterans) are found on trees [49]. Hence, a polydomous arrangement of nests may allow efficient exploitation or resources that occur in regular but isolated patches (trees) interspersed over a harsh environmental matrix (bare ground). Interestingly, results of genetic analysis suggested that single colonies hosted only one queen, irrespective of the number of nests found in the colony. This finding confirms monogyny in *C*. *scutellaris* as suggested by Baroni Urbani and Soulié (see Ref [50]), but also supports the hypothesis that colonies are organized in a “core-satellite” system formed by a queen-hosting nest (the core) and a network of satellite nests [52].

Despite the small size of the study area and the close proximity of nests, little genetic similarity was found among colonies. Additionally, there was no correlation between genetic distance and spatial distance between nests. The sharp genetic segregation recorded among colonies of *C*. *scutellaris* may be a consequence of long distance dispersal strategy, with winged males and queens performing prolonged nuptial flight followed by colony foundation far from the natal nestSuch behavior could indeed determine a clearly defined population genetic structure [76–78]. According to npManova, cuticular chemical profiles varied significantly among colonies and nests. However, the rather complex plots shown by PLS-DA could be the result of a pattern of differentiation among groups (colonies and nests) due to variation of only a few or even a single CHC. Moreover, our results show that variations in the relative proportion of compounds, and consequently their pattern of divergence, could be detected by a few or even a single component. Colony number 8, for instance, clearly segregated from the other colonies based on components 3 and 4 only (Fig 3d). Plots of additional components are reported in S1 File (Figures A-F). The mechanism of hydrocarbon profile assemblage in ants is still a debated issue (see Ref [42] for a review). There is evidence of both a predominant genetic determination (*e*.*g*. *Formica polyctena*, see Ref [29]) and mutual environmental and genetic contribution to the final structure of CHCs profiles (*e*.*g*. *Linepithema humile*, see Refs. [79, 80]). Yet, rearing under controlled laboratory conditions appeared to affect signaling and nest-mate recognition in several ant species (see *e*.*g*. Refs. [36, 81, 82]). Cuticular hydrocarbon profiles can also be heavily affected by diet (see *e*.*g*. Refs. [36, 81]). Our study suggests that signaling system in *Crematogaster scutellaris* is unlikely to depend only upon genetic relatedness, while the role of functional genes and environmental factors needs further investigation. Chemical segregation among highly related nests is particularly interesting for it suggests that a “nest odor” may depend on very local environmental features and on the fact that ants were rarely seen moving from one nest to another within the same cluster [52]. On the other hand, the wide overlap among chemical signatures of different colonies may be due to low variation in the resource spectrum available to ants in xeric agroecosystems.

Aggression among members of different colonies was not as common as expected among unrelated colonies, for it occurred only in approximately 50% of staged tests. Direct comparison with data on other ant species is difficult because of intrinsic methodological differences in staging encounters (e.g. single vs. group encounters) or scoring aggression level (e.g. binary vs. continuous scale). In a number of studies high values of aggressiveness were reported or can be computed from published data (see *e*.*g*. Refs. [83–85]), while lower aggression probability was usually associated with unicolonial and generally invasive species [86–88]. Moreover, we found that aggressive behavior in *C*. *scutellaris* varied considerably. Some colonies were aggressive in less than half of encounters whereas others showed aggressive reactions in the majority of staged tests. Variation in levels of aggressiveness was described both within and between ant colonies. A colony propensity to start a fight was found to vary with nutrients availability [89], productivity [90], presence or absence of a queen [70] and previous fighting experience [91, 92]. We found no correlation between the number of times a colony was involved in aggressive encounters and cluster size (the number of nests found in the colony).

The best model defined by the lowest AICc included chemical, spatial and genetic distances as explanatory variables. Model averaging was necessary as the second-ranked model, which included chemical and spatial distances, received similar support. Although all three variables were retained in the model, chemical and spatial distances had the highest importance, followed by genetic divergence. Interestingly, aggression increased with decreasing of both chemical and spatial distances. These results suggest a “nasty neighbor” behavioral pattern, whereby an individual responds more aggressively against a neighbor than to a distant stranger, for the former is likely to use nearby resources and therefore represent a stronger competitor [93, 94]. A weak positive relationship between aggressiveness and genetic distance was also retained in the final model, even if its effect is low and not significant. Nasty neighbor behavior was described in several ant species, including *Pogonomyrmex barbatus* [37], *Cataglyphis fortis* [95], *Pristomyrmex pungens* [38], *Iridomyrmex purpureus* [96], *Linepithema humile* [97], *Oecophylla smaragdina* [98] and *Formica pratensis* [99]. However, to our knowledge no such behavior was clearly described in the genus *Crematogaster* [53]. Aggression tests were non completely blind (see methods). However, given that the operator was not aware of either genetic or chemical similarity between confronted groups nor had *a priori* expectation on the relationship between aggressiveness and genetic and chemical distances, experimental biases were considered negligible.

Finally, comparison of total cuticular hydrocarbons profile appear to have a relative importance in the analysis of nest-mate recognition in *C*. *scutellaris* ants, since some minor compounds or only a part of the whole chemical signature could be involved in the process [100–102]. Additional studies may investigate a larger array or hydrocarbons and the role that minor hydrocarbons or non-hydrocarbon compounds may play in inter-individual recognition.

In conclusion, this study allowed the description of some important features of the biology and ecology of *C*. *scutellaris*. First, a polydomous arrangement of nests was defined by genetic data and we confirmed the monogyny of colonies in this species. Second, the high level of genetic differentiation among colonies implies a long spatial dispersal strategy during mating period and prior to colony foundation. Finally, we found that *C*. *scutellaris* showed low levels of aggression against non nest-mate conspecifics. However, aggressiveness increased with both spatial proximity and similarity in cuticular hydrocarbon profiles among colonies, suggesting a “nasty neighbor” effect.

## Supporting Information

S1 File PLS-DA plots on chemical distances for genetic clusters (Figures A, B, C) and nests (Figures D, E, F) described by the components (Comp) 5 to 10. Scores were normalised between -1 and 1 and plotted in the same graph with the loadings of variables. Scores of individual ants of the same group are represented by the same number (14 genetic clusters and 29 nests). Loadings of variables are indicated by compound names. Compounds abbreviations: M: methyl; CeM: central-methyl; DM: dimethyl.
(PDF)

S1 Table Summary of spatial (Spatial), genetic (θ-Fst) and chemical (Chem-HC) divergence between each pair of nests from different genetic clusters (GC-1–GC-2) involved in behavioral tests and the number of aggressive individuals recorded (Ag-Indiv). (DOC)
