# Supplementary material for: The Rules of Aggression: How Genetic, Chemical and Spatial Factors Affect Intercolony Fights in a Dominant Species, the Mediterranean Acrobat Ant Crematogaster scutellaris
Source: PLoS One. 2015 Oct 7;10(10):e0137919. doi: 10.1371/journal.pone.0137919 (PMC4596555; doi:10.1371/journal.pone.0137919)
Supplement: S1 File — Scores were normalised between -1 and 1 and plotted in the same graph with the loadings of variables. Scores of individual ants of the same group are represented by the same number (14 genetic clusters and 29 nests). Loadings of variables are indicated by compound names. Compounds abbreviations: M: methyl; CeM: central-methyl; DM: dimethyl. (PDF) [file pone.0137919.s001.pdf]

S1 File. PLS-DA plots on chemical distances for genetic clusters (Figures A, B, C) and nests (Figures D, E, F) described by the components (Comp) 5 to 10. Scores were normalised between -1 and 1 and plotted in the same graph with the loadings of variables. Scores of individual ants of the same group are represented by the same number (14 genetic clusters and 29 nests). Loadings of variables are indicated by compound names. Compounds abbreviations: M: methyl; CeM: central-methyl; DM: dimethyl.

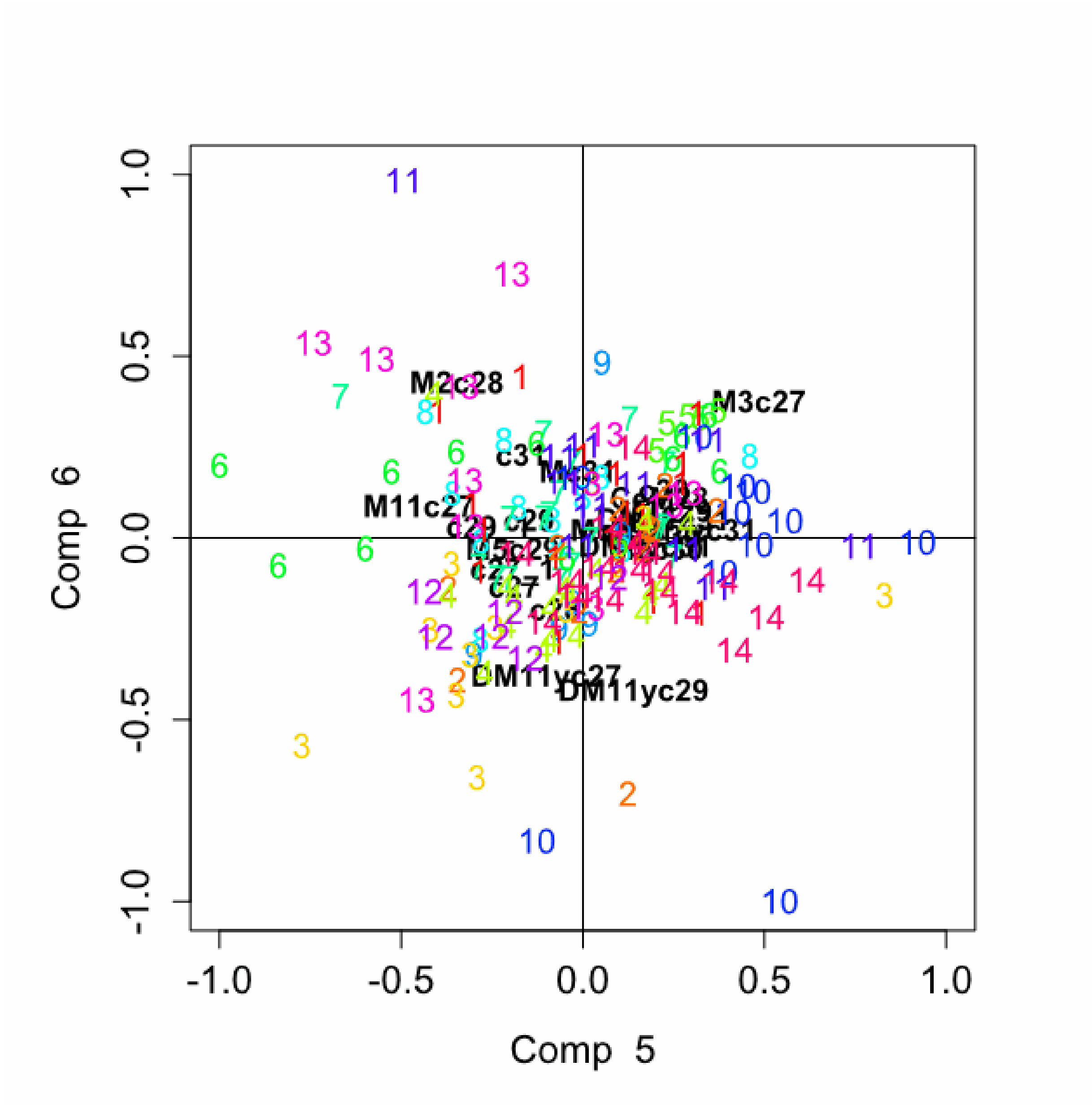
Comp 6
Comp 5
1.0
0.5
0.0
-0.5
-1.0
-1.0
-0.5
0.0
0.5
1.0
M2c28
M3c27
M11c27
DM11yc27
DM11yc29

Figure A.

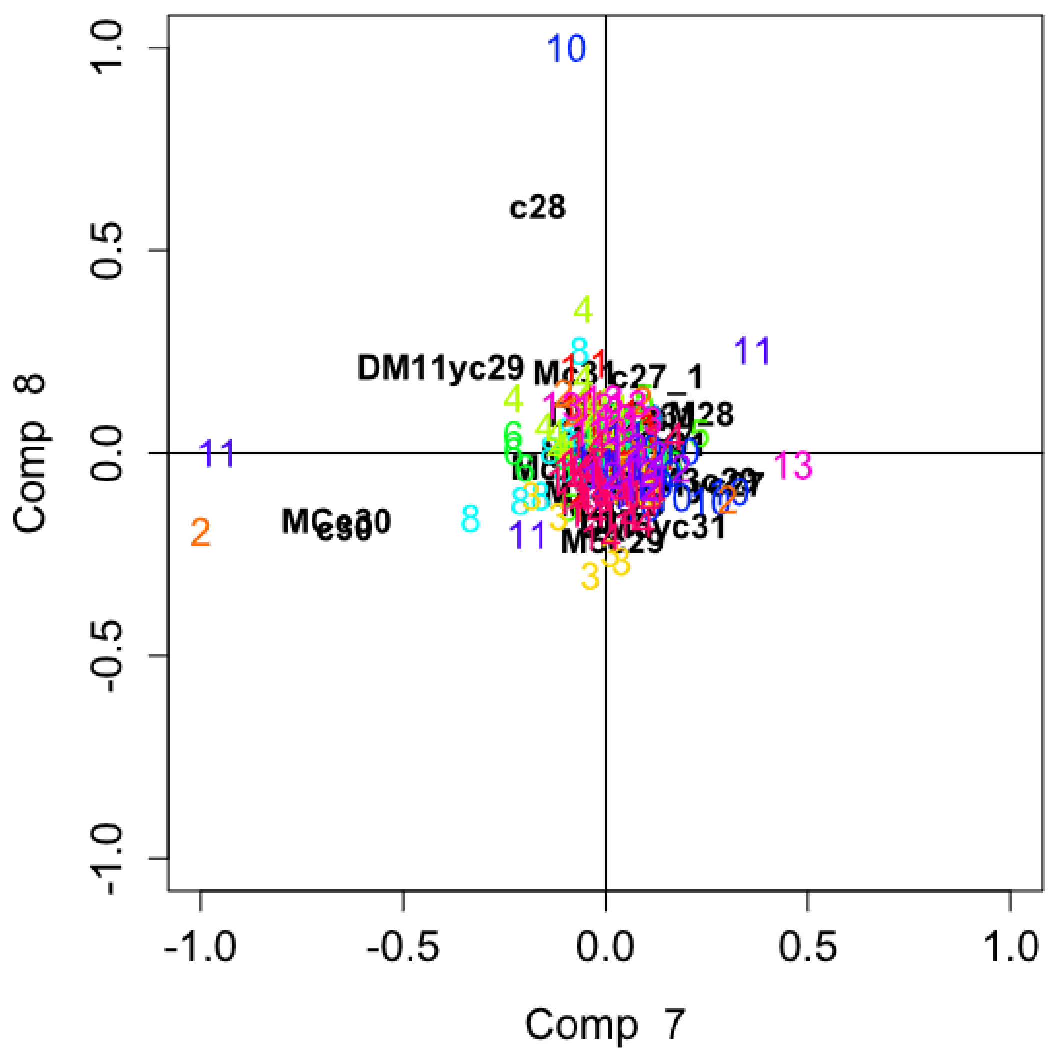

Figure B.

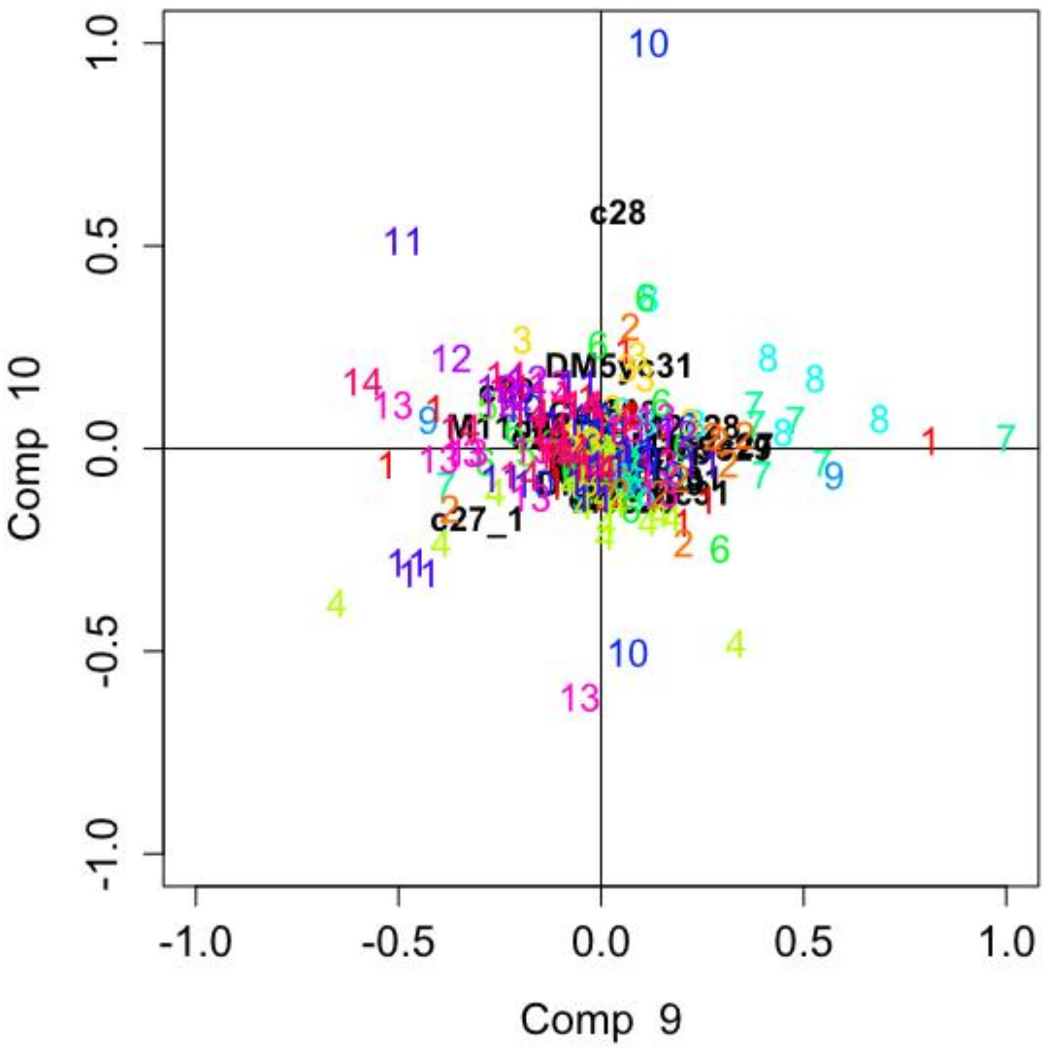

Figure C.

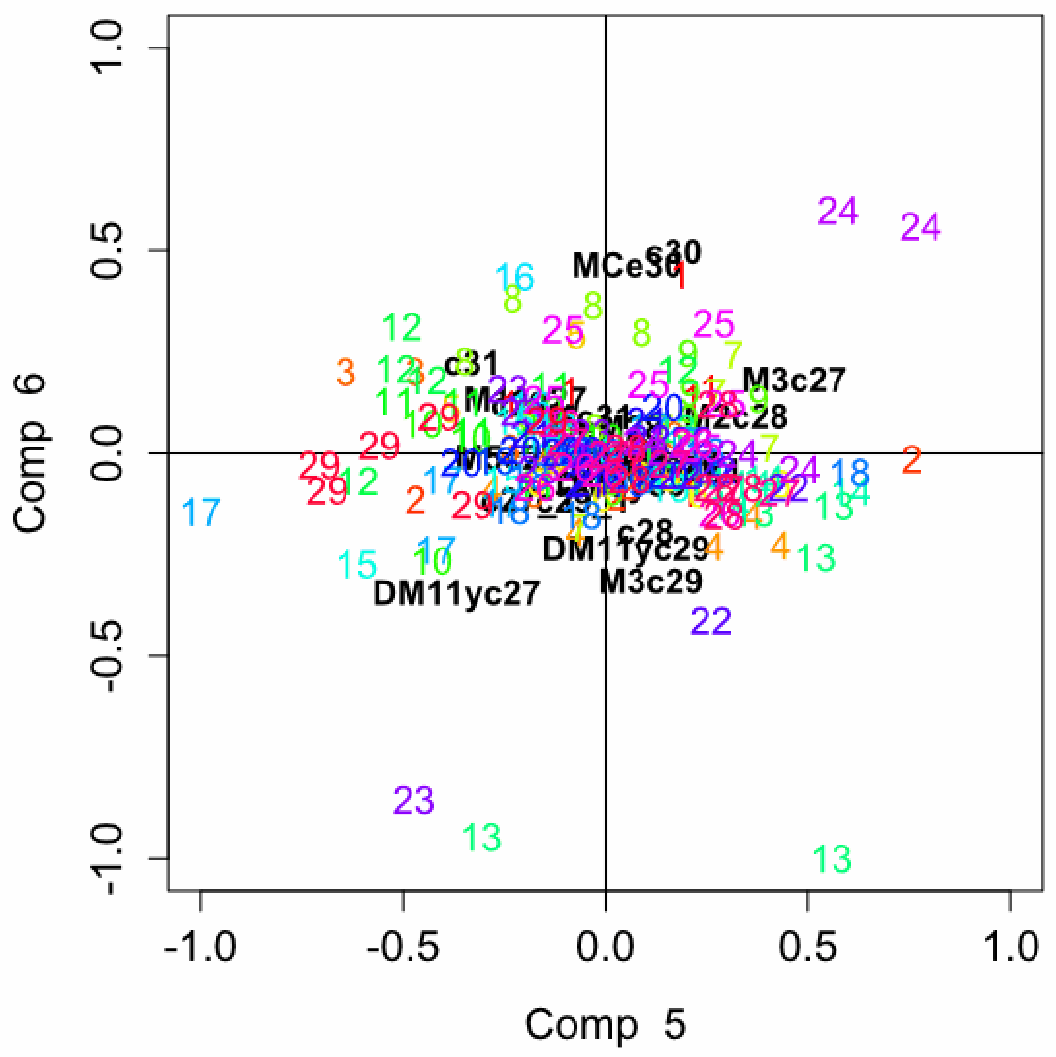
Comp 6
1.0
0.5
0.0
-0.5
-1.0
Comp 5
-1.0
-0.5
0.0
0.5
1.0
c31
M3c27
c28
DM11yc27
M3c29

Figure D.

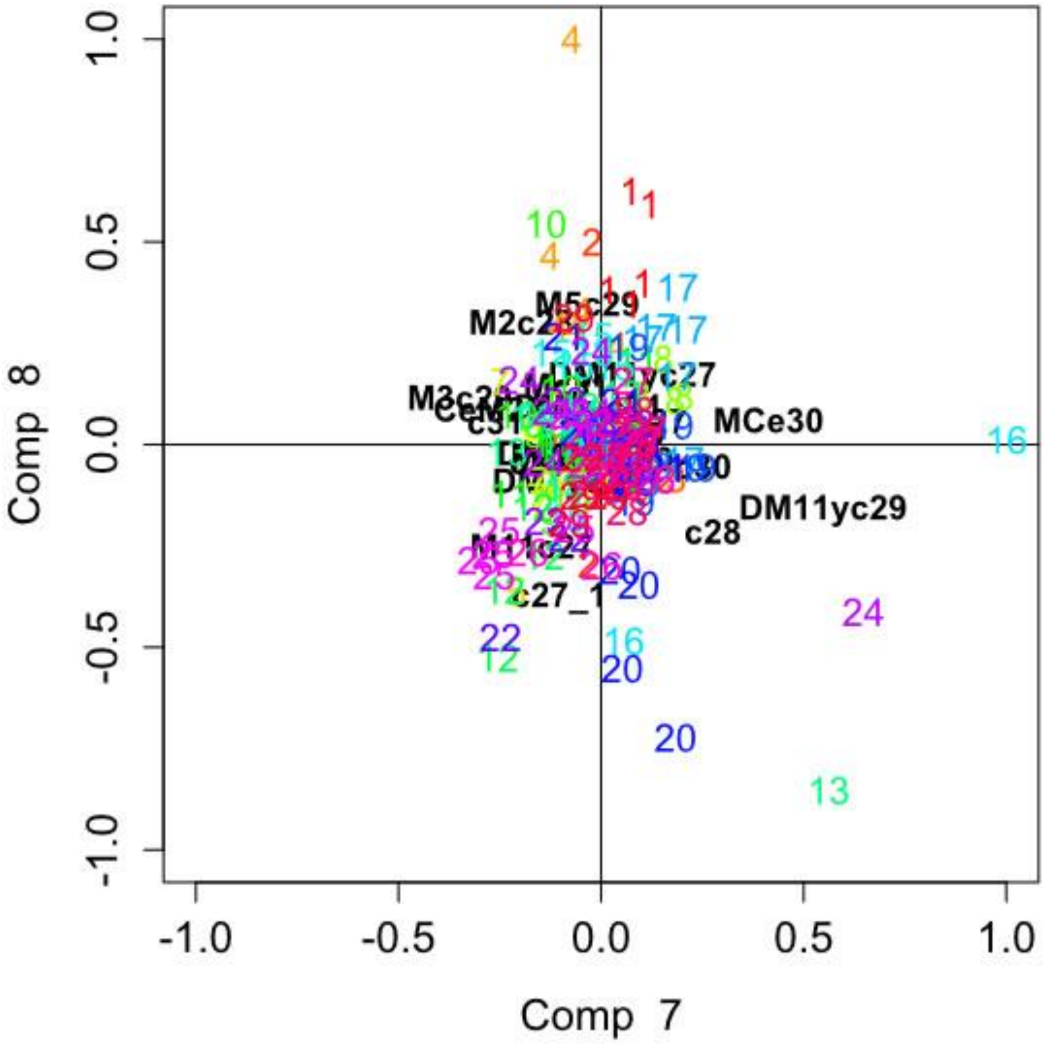

Figure E.

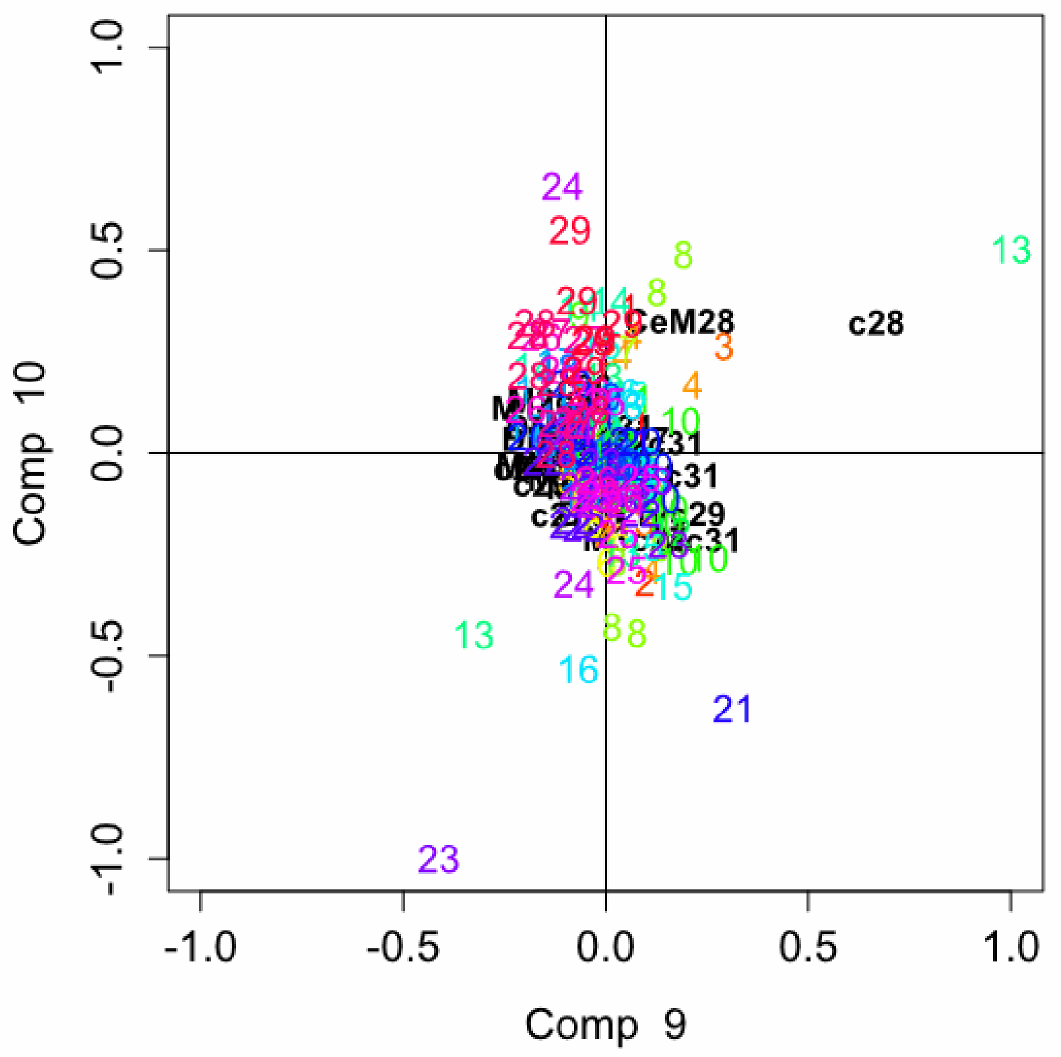

Figure F.
