# Supplementary material for: The Rules of Aggression: How Genetic, Chemical and Spatial Factors Affect Intercolony Fights in a Dominant Species, the Mediterranean Acrobat Ant Crematogaster scutellaris
Source: PLoS One. 2015 Oct 7;10(10):e0137919. doi: 10.1371/journal.pone.0137919 (PMC4596555; doi:10.1371/journal.pone.0137919)
Supplement: S1 Table — (DOC) [file pone.0137919.s002.doc]

**S1 Table.** Summary of spatial (Spatial), genetic (*θ*-Fst) and chemical (Chem-HC) divergence between each pair of nests from different genetic clusters (GC-1 - GC-2) involved in behavioral tests and the number of aggressive individuals recorded (Ag-Indiv).

| **GC-1** | | **GC-2** | | **Spatial** | ***θ*-Fst** | **Chem-HC** | | **Ag-Indiv** |
| --- | --- | --- | --- | --- | --- | --- | --- | --- |
| 1 | 2 | | 45.810 | | 0.357 | 0.269 | 9 | |
| 1 | 3 | | 52.068 | | 0.339 | 0.200 | 0 | |
| 1 | 4 | | 91.857 | | 0.360 | 0.191 | 0 | |
| 1 | 5 | | 97.717 | | 0.386 | 0.335 | 5 | |
| 1 | 6 | | 97.513 | | 0.361 | 0.279 | 0 | |
| 1 | 7 | | 52.983 | | 0.430 | 0.195 | 8 | |
| 1 | 8 | | 81.906 | | 0.362 | 0.205 | 7 | |
| 1 | 9 | | 77.947 | | 0.406 | 0.213 | 2 | |
| 1 | 10 | | 24.862 | | 0.402 | 0.232 | 0 | |
| 1 | 11 | | 67.341 | | 0.453 | 0.236 | 7 | |
| 1 | 12 | | 56.908 | | 0.426 | 0.196 | 6 | |
| 1 | 13 | | 78.382 | | 0.396 | 0.207 | 6 | |
| 1 | 14 | | 48.551 | | 0.414 | 0.182 | 7 | |
| 2 | 3 | | 11.572 | | 0.345 | 0.252 | 9 | |
| 2 | 4 | | 46.872 | | 0.323 | 0.288 | 0 | |
| 2 | 5 | | 99.563 | | 0.412 | 0.226 | 0 | |
| 2 | 6 | | 95.358 | | 0.333 | 0.241 | 7 | |
| 2 | 7 | | 65.522 | | 0.444 | 0.272 | 6 | |
| 2 | 8 | | 99.183 | | 0.414 | 0.270 | 0 | |
| 2 | 9 | | 94.426 | | 0.361 | 0.297 | 0 | |
| 2 | 10 | | 46.807 | | 0.425 | 0.290 | 0 | |
| 2 | 11 | | 107.060 | | 0.467 | 0.291 | 9 | |
| 2 | 12 | | 94.179 | | 0.405 | 0.240 | 5 | |
| 2 | 13 | | 105.779 | | 0.356 | 0.265 | 5 | |
| 2 | 14 | | 77.123 | | 0.422 | 0.260 | 0 | |
| 3 | 4 | | 45.976 | | 0.400 | 0.172 | 0 | |
| 3 | 5 | | 91.367 | | 0.392 | 0.324 | 0 | |
| 3 | 6 | | 86.526 | | 0.349 | 0.256 | 8 | |
| 3 | 7 | | 61.241 | | 0.424 | 0.174 | 6 | |
| 3 | 8 | | 94.297 | | 0.409 | 0.182 | 9 | |
| 3 | 9 | | 89.514 | | 0.329 | 0.182 | 3 | |
| 3 | 10 | | 56.947 | | 0.396 | 0.212 | 3 | |
| 3 | 11 | | 108.441 | | 0.419 | 0.210 | 1 | |
| 3 | 12 | | 94.978 | | 0.427 | 0.180 | 7 | |
| 3 | 13 | | 116.678 | | 0.331 | 0.195 | 6 | |
| 3 | 14 | | 87.609 | | 0.429 | 0.162 | 7 | |
| 4 | 5 | | 130.042 | | 0.417 | 0.387 | 0 | |
| 4 | 6 | | 123.552 | | 0.301 | 0.299 | 0 | |
| 4 | 7 | | 106.431 | | 0.405 | 0.166 | 0 | |
| 4 | 8 | | 138.562 | | 0.347 | 0.173 | 0 | |
| 4 | 9 | | 133.810 | | 0.345 | 0.157 | 4 | |
| 4 | 10 | | 86.099 | | 0.452 | 0.207 | 0 | |
| 4 | 11 | | 153.633 | | 0.476 | 0.200 | 5 | |
| 4 | 12 | | 140.465 | | 0.438 | 0.202 | 4 | |
| 4 | 13 | | 136.636 | | 0.340 | 0.203 | 0 | |
| 4 | 14 | | 112.379 | | 0.391 | 0.150 | 0 | |
| 5 | 6 | | 8.814 | | 0.352 | 0.218 | 0 | |
| 5 | 7 | | 45.004 | | 0.474 | 0.352 | 0 | |
| 5 | 8 | | 33.580 | | 0.404 | 0.336 | 0 | |
| 5 | 9 | | 32.666 | | 0.379 | 0.388 | 0 | |
| 5 | 10 | | 120.780 | | 0.422 | 0.357 | 0 | |
| 5 | 11 | | 89.672 | | 0.483 | 0.349 | 1 | |
| 5 | 12 | | 78.881 | | 0.412 | 0.299 | 4 | |
| 5 | 13 | | 174.971 | | 0.375 | 0.325 | 0 | |
| 5 | 14 | | 145.918 | | 0.383 | 0.343 | 6 | |
| 6 | 7 | | 45.965 | | 0.361 | 0.282 | 0 | |
| 6 | 8 | | 40.635 | | 0.342 | 0.278 | 6 | |
| 6 | 9 | | 38.996 | | 0.222 | 0.312 | 0 | |
| 6 | 10 | | 119.746 | | 0.379 | 0.299 | 0 | |
| 6 | 11 | | 95.344 | | 0.408 | 0.299 | 0 | |
| 6 | 12 | | 83.848 | | 0.395 | 0.252 | 0 | |
| 6 | 13 | | 175.483 | | 0.327 | 0.261 | 0 | |
| 6 | 14 | | 146.012 | | 0.332 | 0.268 | 0 | |
| 7 | 8 | | 33.731 | | 0.399 | 0.180 | 4 | |
| 7 | 9 | | 29.015 | | 0.348 | 0.174 | 5 | |
| 7 | 10 | | 76.756 | | 0.465 | 0.208 | 0 | |
| 7 | 11 | | 60.333 | | 0.486 | 0.200 | 4 | |
| 7 | 12 | | 46.493 | | 0.421 | 0.199 | 0 | |
| 7 | 13 | | 129.980 | | 0.358 | 0.202 | 0 | |
| 7 | 14 | | 100.946 | | 0.488 | 0.165 | 7 | |
| 8 | 9 | | 4.783 | | 0.293 | 0.184 | 10 | |
| 8 | 10 | | 106.682 | | 0.403 | 0.215 | 3 | |
| 8 | 11 | | 57.062 | | 0.455 | 0.207 | 0 | |
| 8 | 12 | | 47.840 | | 0.349 | 0.217 | 2 | |
| 8 | 13 | | 154.036 | | 0.297 | 0.216 | 0 | |
| 8 | 14 | | 126.969 | | 0.309 | 0.169 | 0 | |
| 9 | 10 | | 102.653 | | 0.405 | 0.211 | 7 | |
| 9 | 11 | | 57.068 | | 0.471 | 0.192 | 6 | |
| 9 | 12 | | 46.905 | | 0.368 | 0.227 | 6 | |
| 9 | 13 | | 150.934 | | 0.322 | 0.224 | 0 | |
| 9 | 14 | | 123.534 | | 0.313 | 0.169 | 7 | |
| 10 | 11 | | 88.239 | | 0.492 | 0.221 | 6 | |
| 10 | 12 | | 79.525 | | 0.442 | 0.244 | 0 | |
| 10 | 13 | | 60.603 | | 0.377 | 0.236 | 3 | |
| 10 | 14 | | 30.813 | | 0.449 | 0.195 | 5 | |
| 11 | 12 | | 14.144 | | 0.489 | 0.257 | 5 | |
| 11 | 13 | | 115.245 | | 0.431 | 0.239 | 7 | |
| 11 | 14 | | 94.922 | | 0.524 | 0.197 | 0 | |
| 12 | 13 | | 113.513 | | 0.318 | 0.192 | 0 | |
| 12 | 14 | | 90.379 | | 0.458 | 0.187 | 7 | |
| 13 | 14 | | 30.243 | | 0.403 | 0.188 | 0 | |
